# Supplementary material for: Identification of photoperiod-induced specific miRNAs in the adrenal glands of Sunite sheep (Ovis aries)
Source: Front Vet Sci. 2022 Jul 22;9:888207. doi: 10.3389/fvets.2022.888207 (PMC9354845; doi:10.3389/fvets.2022.888207)
Supplement: Supplementary file 1 [file Data_Sheet_1.ZIP › Supplementary file/miRDeep2 file.zip!/miRDeep2 file/Know_Structure/SP42A3/oar-mir-127.pdf]

miRBase precursor : oar-mir-127
Total read count : 74626
oar-miR-127 read count : 73282
remaining reads : 1344

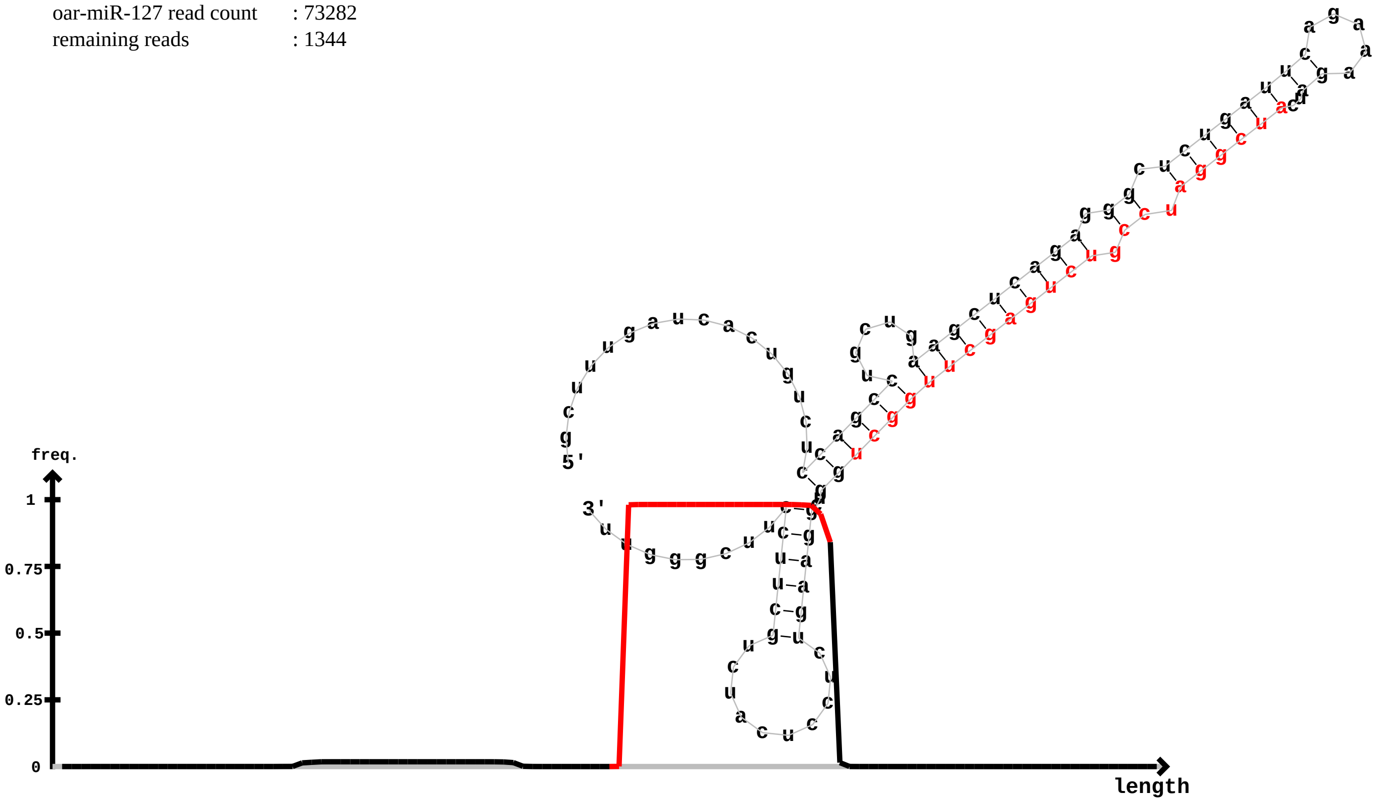

oar-miR-127

```
5'- gcuuugaucacugucuccagccugcugaagcucagagggcucugauucagaaagaucaucggauccgucugagcuuggcuggucggaagucuccucaucugcuuccuucggguu   -3'   exp
    ................((((((.....(((((((((.((.((((((((.....))..)))))).)).)))))))))))))))..((((((..........))))))........   reads   mm   sample
    gcuuugaucacugucuccagccug..........................................................................................   1   0   seq
    .cuuugaucacugucuccagccug..........................................................................................   1   0   seq
    ..uuugaucacugucuccagccug..........................................................................................   2   0   seq
    ...uugaucacugucuccagccug..........................................................................................   2   0   seq
    ......................ugcugaagcucagagggcucug......................................................................   3   0   seq
    ......................ugcugaagcucagagggcucuga.....................................................................   7   0   seq
    ......................ugcugaagcucagagggcucugau....................................................................   6   0   seq
    .......................gcugaagcucagagggcucuga.....................................................................   3   0   seq
    .......................gcugaagcucagagggcucugau....................................................................   5   0   seq
    ........................cugaagcucagagggcuc........................................................................   1   0   seq
    ........................cugaagcucagagggcucug......................................................................   2   0   seq
    ........................cugaagcucagagggcucuga.....................................................................   32   0   seq
    ........................cugaagcucagagggcucugau....................................................................   171   0   seq
    ........................cugaagcucagagggcucugauu...................................................................   773   0   seq
    ........................cugaagcucagagggcucugauuc..................................................................   17   0   seq
    .........................ugaagcucagagggcucug......................................................................   1   0   seq
    .........................ugaagcucagagggcucuga.....................................................................   1   0   seq
    .........................ugaagcucagagggcucugau....................................................................   6   0   seq
    .........................ugaagcucagagggcucugauu...................................................................   154   0   seq
    .........................ugaagcucagagggcucugauuc..................................................................   2   0   seq
    ..........................gaagcucagagggcucugau....................................................................   1   0   seq
    ..........................gaagcucagagggcucugauu...................................................................   92   0   seq
    ..........................gaagcucagagggcucugauuc..................................................................   51   0   seq
    ..........................gaagcucagagggcucugauuca.................................................................   2   0   seq
    ..........................gaagcucagagggcucugauucag................................................................   1   0   seq
    ...........................aagcucagagggcucuga.....................................................................   1   0   seq
    ...........................aagcucagagggcucugauu...................................................................   4   0   seq
    ...........................aagcucagagggcucugauucag................................................................   1   0   seq
    ...........................aagcucagagggcucugauucaga...............................................................   1   0   seq
    .......................................................ucaucggauccgucugagcuuggcu..................................   2   0   seq
    ........................................................caucggauccgucugagcuugg....................................   1   0   seq
    .........................................................aucggauccgucugagcuugg....................................   7   0   seq
    .........................................................aucggauccgucugagcuuggc...................................   21   0   seq
    .........................................................aucggauccgucugagcuuggcu..................................   4   0   seq
    ..........................................................ucggauccgucugagcu.......................................   4   0   seq
```

oar-miR-127

```
gcuuugaucacugucuccagccugcugaagcucagagggcucugauucagaaagaucaucggauccgucugagcuuggcuggucggaagucuccucaucugcuuccuucggguu
..........................................................ucggauccgucugagcuu......................................    98      0    seq
..........................................................ucggauccgucugagcuug.....................................    142     0    seq
..........................................................ucggauccgucugagcuugg....................................    2424    0    seq
..........................................................ucggauccgucugagcuuggc...................................    7829    0    seq
..........................................................ucggauccgucugagcuuggcu..................................    61569   0    seq
..........................................................ucggauccgucugagcuuggcug.................................    1073    0    seq
..........................................................ucggauccgucugagcuuggcugg................................    16      0    seq
...........................................................cggauccgucugagcuu......................................    1       0    seq
...........................................................cggauccgucugagcuug.....................................    1       0    seq
...........................................................cggauccgucugagcuugg....................................    4       0    seq
...........................................................cggauccgucugagcuuggc...................................    7       0    seq
...........................................................cggauccgucugagcuuggcu..................................    64      0    seq
...........................................................cggauccgucugagcuuggcug.................................    6       0    seq
............................................................ggauccgucugagcuuggc...................................    1       0    seq
............................................................ggauccgucugagcuuggcu..................................    8       0    seq
```
